# Characterization of Hybrid Epoxy Nanocomposites

**DOI:** 10.3390/nano2040348

**Published:** 2012-10-26

**Authors:** Shelly Simcha, Ana Dotan, Samuel Kenig, Hanna Dodiuk

**Affiliations:** Shenkar College of Engineering and Design, Ramat Gan, 52562, Israel; Email: shellysimcha@yahoo.com (S.S.); samkenig@shenkar.ac.il (S.K.); hannad@shenkar.ac.il (H.D.)

**Keywords:** nanocomposites, carbon nanotubes, epoxy, sol-gel

## Abstract

This study focused on the effect of Multi Wall Carbon Nanotubes (MWCNT) content and its surface treatment on thermo-mechanical properties of epoxy nanocomposites. MWCNTs were surface treated and incorporated into two epoxy systems. MWCNT's surface treatments were based on: (a) Titania coating obtained by sol-gel process and (b) a nonionic surfactant. Thermo-mechanical properties improvement was obtained following incorporation of treated MWCNT. It was noticed that small amounts of titania coated MWCNT (0.05 wt %) led to an increase in the glass transition temperature and stiffness. The best performance was achieved adding 0.3 wt % titania coated MWCNT where an increase of 10 °C in the glass transition temperature and 30% in storage modulus were obtained.

## 1. Introduction

Carbon Nanotubes (CNTs) have high potential to improve the properties of polymers. Due to their unique structure and high aspect ratio (length to diameter ratio, L/D), they possess an outstanding combination of exceptional mechanical, electrical and thermal properties in addition to flexibility and low density. Nevertheless, CNTs give rise to aggregation due to their high aspect ratio, flexibility, strong inter-tube Van Der Waals (VDW) attraction. The key factors for properties improvement of polymer/CNT nanocomposites are good dispersion and low agglomeration [1,2,3,4]. Consequently, two approaches have been proposed to disperse CNT in polymer matrix: application of stresses to break mechanically the agglomerates and obtain adequate dispersion and surface treatments of CNT based on chemical and physical methods [5,6,7,8,9,10]. According to Coleman *et al.* [3] well dispersed CNT is of utmost importance to maximize the interfacial area, to achieve efficient load transfer and minimize the presence of stress concentration centers. The frictional forces in polymer/CNT interfaces are expected to be large and, even if debonding has occurred, there will be significant load transfer from the polymer to the CNT, contrary to traditional fiber reinforced composite, where complete debonding implies failure of the composite [11,12,13,14]. According to Huang *et al*. [14] good dispersion can be seen at low concentrations of CNT and they re-aggregate after long period of time. At concentrations above the threshold (2-3 wt %) an elastic gel of entangled CNT has been observed. Furthermore, high concentration of CNT in epoxy increases resin viscosity and makes the dispersion of CNT extremely difficult [15]. Compatible surface treatment of CNT with the epoxy matrix has been found to give good stress transfer at the interface [4]. An effective dispersion of CNT requires overcome inter-tube attraction, separating CNT from aggregates and stabilizing them within the polymer matrix. In order to optimize the dispersion of CNTs in a polymer matrix chemical surface treatments can be used together with mechanical dispersion methods (ultrasonication, calendering, ball milling and shear mixing) [10,11,12,13,14,15,16,17]. Direct covalent treatment is associated with a change of hybridization from sp^2^ to sp^3^ and a simultaneous loss of ð-conjugation system in CNT. This is achieved by processes such as: fluorination, hydrogenation, cyclo-addition, oxidation and further derivative reactions. Defect sites on CNT surface like: open ends, holes in the sidewalls, irregularities in CNT scaffold, are utilized in order to tie up different functional groups. Oxidation can be obtained by wet chemical methods with concentrated acids or by milder approaches using the photo-oxidation, oxygen plasma, or gas phase treatment. [10,18,19,20,21,22,23,24,25,26,27,28,29,30]. Covalent treatments greatly enhance the solubility of CNT and can provide multiple and strong bonding sites to the polymer matrix. It is believed that the improvements in nanocomposite properties are addressed in connection with improved dispersion and efficient load transfer via epoxy/CNT interface. However, this route can strongly influence the intrinsic characteristics of CNT and result in fragmentation and degradation of CNT structure, which further reflects in the overall performance of polymer/CNT nanocomposites [7,15].

Table 1 summarizes the main literature based experimental results and emphasizes the effect of covalent treatment for MWCNT on the thermo-mechanical properties of epoxy nanocomposites. It can be noticed that small amounts of covalent treated MWCNT (in most cases less than 1 wt %) have a positive effect on thermo-mechanical properties of epoxy nanocomposites. The results show an average increase of 40%-50% in modulus and strength and an average increase of 20% of Tg, compared to neat epoxy. Covalent treated MWCNT also improved the toughness of nanocomposites. In addition, amino-functionalization seems to be a usual surface treatment of CNT due to its high reactivity with epoxy resin [29,30,31,32,33,34,35,36,37,38,39]. Non-covalent treatments are an efficient alternative route to functionalized CNT and yet preserving the intrinsic characteristics of CNT and ð-conjugation system. In addition, this route leads to higher efficiency of the functionalization, compare to covalent treatments where VDW active sites on CNT surface are limited (mostly at the defects and end caps). There are three main methods for non-covalent CNT treatments: (a) polymer wrapping; (b) surfactant adsorption and (c) endohedral method. Polymer wrapping process is achieved through the interactions and ð-ð stacking between CNT and polymer chains containing aromatic rings. The surfactants studied are classified according to the charge of their head groups: nonionic, anionic and cationic. In general, ionic surfactants have some advantages in water-soluble polymers, while nonionic surfactants are proposed in case of water-insoluble polymers. There are three possible surfactant-CNT interaction mechanisms: (a) electrostatic attraction or repulsion; (b) hydrogen bonding between the -O- of polyethoxylate and -OH and/or -COOH groups of CNT and; (c) hydrophobic and π-π interactions. According to Vaisman *et al.* [8] it is important to achieve mechanical dispersion of CNT prior to surfactant adsorption in order to obtain an efficient de-agglomeration of CNT. In the “endohedral” method guest atoms or molecules are inserted in the inner cavity of CNT through defect sites localized at the ends or on the sidewalls [10,39,40]. Surfactants composed of hydrophilic polyethoxyl group (including polyethoxyl chain and hydroxyl groups) and hydrophobic group (4-(1,1,1,3-tetramethylbutyl)-phenyl group) bridge between CNT and polymers [39,40]. According to Bai *et al.* [40] adsorption of Triton X-series (*i.e.*, Triton X-305, Triton X-165, Triton X-114, Triton X-100) surfactants facilitates the suspension of MWCNT in water mainly by hydrophobic and π-π interactions mechanisms. Furthermore, Triton X-series with shorter hydrophilic chains showed higher suspendability of MWCNT, which could be affected by the surfactant adsorption and, also possibly through the formation of larger micelles both in surfactant solution and on MWCNT surface at the surfactant concentration above the CMC. Rastogi *et al.* [41] investigated the dispersion of MWCNT in aqueous medium using four different surfactants. It was concluded that Triton X-100 (Polyoxyethylene octyl phenyl ether) has the highest dispersing power by virtue of its benzene ring. Molecules having a benzene ring structure adsorb more strongly to the graphitic surface due to π-π stacking type interaction. In addition, MWCNT-to surfactant ratio has shown to affect the MWCNT dispersion significantly. The poor dispersion at high surfactant loading was concluded to be caused by a possible mechanism of flocculation. Flocculation of CNT occurs when portions of surfactant molecules interact with others surfactant molecules on neighboring CNT, thus bridging between CNT and avoid dispersion. Coating of CNT surface is an additional method based on non-covalent treatments. CNT was coated with a precursor of titania by sol-gel or hydrothermal methods [42,43,44,45]. In order to improve the affinity of titania coated CNT to a polymer it can be modified with a coupling agent such as silane. *Sol-gel* coatings can be obtained by a simple process based on hydrolysis and polycondensation reactions carried usually at room temperature [42,43,44,45,46,47].

Table 2 summarizes the main literature based experimental results emphasizing the effect of different non-covalent treated MWCNT on thermo-mechanical properties of epoxy nanocomposites. It can be concluded that small amounts of non-covalent treated MWCNT (1 wt %) have a positive effect on thermo-mechanical properties of epoxy nanocomposites. An average increase of 50%-60% in modulus and strength and 20% in Tg could be noticed. Non-covalent treated MWCNT also improved the toughness of nanocomposites [48,49,50,51,52,53,54,55,56].

**Table 1:** Effect of covalent treatment of MWCNT on the thermo-mechanical properties of epoxy nanocomposites.

Covalent treatment	Mechanical dispersion	Solvent dispersion	Content (wt %)	Maximum increase in thermo-mechanical properties compared to neat epoxy (%)					Remarks	Ref.
				ModulusS,T,F	StrengthT,F	Tg	Toughness			
Amino groups	Stirred, sonicated		0.01-1	18.95F	120.41F	10.53			Aromatic and less aliphatic amino group structure is better for mechanical reinforcement.	15,28
	Sonicated		0.05-0.75			29.69			Suspension of MWCNT was performed within the hardener.	29
	Stirred, sonicated	Acetone	0.1-2		51T	22.38	93 (kJ/m^2^)		Amino groups can act as a curing agent and lead to a more highly cross-linked structure.	30
	Stirred			53S	26.32F	5.34			Amine functionalization improved curing kinetics. MWCNT content was 3 phr of epoxy resin.	31
TETA Tri ethylene tetra amine	Stirred, sonicated	Ethyl alcohol	0.2-1	22F	29F		84 (kJ/m^2^)			32
	Stirred, sonicated		0.05-0.5			18.64	97 (kJ/m^2^)		Epoxy contain 0.05 wt % TETA-MWCNT kept near 50% light transmittance of the neat epoxy.	33
Polyetheramine (JeffamineT-403)	Stirred, sonicated	Chloroform	1	27.51T	104.17T				Mechanical properties of nanocomposites based rubbery system (Tg = 25 °C) was higher compared to glassy system (Tg = 80 °C). Suspention of MWCNT was performed within the hardener.	16
Oxidation	Sonicated	Acetone	1	75S (HNO_3_ treatment)					The increase of surface oxygen per type of treatment is: HCl< NH_4_OH/H_2_O_2_ < piranha < refluxed HNO_3_. Higher degree of oxidation leads to higher degradation of the CNT shell.	27
Plasma oxidation	Sonicated		1	2. 33T	123T			Nanocomposites containing plasma treated MWCNT showed the best mechanical and rheological behavior, compared to those containing acid and amine treated MWCNT.		34
Plasma maleic anhydride	Shear mixed, sonicated		0.1-1	100T	50T	27		Suspension of MWCNT was performed within the hardener.		35
Silane	Sonicated	Ethanol	0.05-0.5	24.14F	14.40F	8.84	10.17(MPa m^2^)			36
Epoxy	Stirred, sonicated	Acetone	0.1-1	90T	45T	16.67				37
PEI	Stirred, sonicated	Acetone	1					PEI was covalently and non-covalently attached to MWCNT. Covalently treated MWCNT exhibited higher storage modulus compare to non-covalently treated and untreated MWCNT.		38

S for storage, T for tensile and F for flexural.

**Table 2:** Effect of non-covalent treated MWCNT on the thermo-mechanical properties of epoxy nanocomposites.

Non-covalent treatment	Mechanical dispersion	Solvent dispersion	Content (wt %)	Maximum increase in thermo-mechanical properties compared to neat epoxy (%)				Remarks	Ref.
				ModulusS,T,F	StrengthT,F	Tg	Toughness		
Sodium salt of 2-aminoethanol	Sonicated		0.1	26S				Improvement in electrical conductivity with a percolation threshold of 0.05 wt %	48
Triton X-100	Sonicated	Acetone	0.025-0.25	24.14F	17.92F	27.59	51.51 (kJ/m^2^)	Triton X-100 (10 CMC) treatment showed the best performance.	39
Silane modified Titania coating		Acetone	0.25-1	85.03F 58.57T	93.04F 115.90T				45
Polyaniline coating	Sonicated	Toluene	0.5-10	52T 150S	61T			There is an optimal value of polyaniline concentration above which mechanical properties of nanocomposites decreased.	49
BCP (Disperbyk-2150)	Stirred, sonicated	Ethanol	0.25	50T	50T			The BCP act as dispersing agent: the lyophobic (solvent repelling) blocks adsorb onto the surface of CNT, while the lyophilic (solvent attracting) blocks are swollen by the solution.	50,51
			0.5-1			23.21			
Titania coating	Homogenized		0.05-0.3	31.8S				Previous study. Tan delta curves below the Tg of nanocomposites showed increase in toughness without dependency in MWCNT treatment.	
	Sonicated		0.05-0.3	27S		10		Current study. MWCNT treated with Triton X-100 didn’t show thermo-mechanical improvement.	

S for storage, T for tensile and F for flexural.

**Table 3:** Epoxy systems [57,58,59].

Epoxy system	Molecular structure			Tg (°C)
	Resin	Curing agent	Diluent	
1. Epon 828/Jeffamine T-403 (5/2)	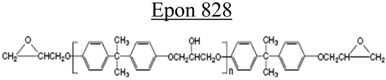	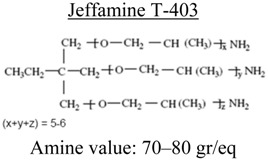		~70
2. LY556/Amine hardener A/DY026(20/5/2)	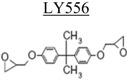	Amine hardener A Confidential Amine value: 72-90 gr/eq	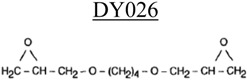	~150

In the current study, Multi Wall Carbon Nanotubes (MWCNT) were surface treated and incorporated into epoxy matrix. One of the MWCNT treatments was based on a titania (TiO_2_) coating obtained by sol-gel process. The surfactant Polyoxyethylene octyl phenyl ether (Triton X-100) was also used as a surface treatment. The study focused on the influence of MWCNT content and surface treatment on thermo-mechanical properties of the nanocomposites. The objective of this work was to characterize the properties of epoxy CNT nanocomposites based to be used as composite's matrix for filament winding technology. The main factor in selecting a resin for filament winding is adequate viscosity. Low viscosity is desirable to help wet the fibers, spread the fibers and lower the friction over the guides during the winding process.

## 2. Experimental

### 2.1. Materials

The epoxy systems that have been utilized were based on diglycidyl ether of bis-phenol A (DGEBPA) and detailed in Table 3.

MWCNTs have been incorporated into the two different epoxy systems. MWCNTs were coated with titania and Triton X-100 (Table 4). Titania coating was obtained according to procedure of Nemeth *et al.* [42] through the dispersion of 0.5 gr of MWCNT in 500 gr of isopropanol; 1.5 gr of titanium (IV) butoxide was added to the resulting dispersion; 250 gr of distilled water was added into the mixture; and the resulting mixture was stirred for 48 h; the MWCNT were filtered, dried, and calcined at 300 °C for 1 h. The surface treatment with Triton X-100 was obtained by dispersing MWCNT with Triton X-100 in ethanol, the resulting mixture was mechanically blended for 10 h and ultrasonic mixed for 1 h, the mixture was left for 36 h at room temperature, for swelling and dried for 8 h at 80 °C. Nanoparticles were dispersed in the resin (Part A), and sonicated for 10 min. Curing agent was added and degassing was applied using a vacuum system for 3 h. The resin systems were cured according to manufacturers prescribed requirements.

**Table 4:** List of materials [60,61,62,63].

Materials	Manufacturer	Chemical name	Molecular structure
MWCNT NC7000	Nanocyl		
Precursor of titania	Sigma-Aldrich	titanium(IV) butoxide	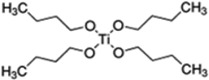
Triton X-100	Sigma-Aldrich		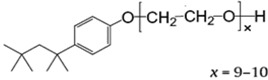

### 2.2. Characterization Methods

Rheology properties were measured using a Dynamic Parallel Plate Rheometer (TA AR2000) using an oscillatory shear mode at a temperature range of 25-70 °C, constant strain of 2%, frequency of 1 Hz and heating rate of 5 °C/min. Thermo-mechanical properties were measured using a DMA analyzer (TA Q800) using single cantilever and three point bending mode, constant amplitude of 15 µm, at a heating rate of 2 °C/min and a frequency of 1 Hz. Microscopy images were obtained using contact mode AFM (Autoprobe CP Research, ThermoMicroscopes) and SEM (Zeiss ULTRA 55, Oberkochen).

## 3. Results and Discussion

**Figure 1:**
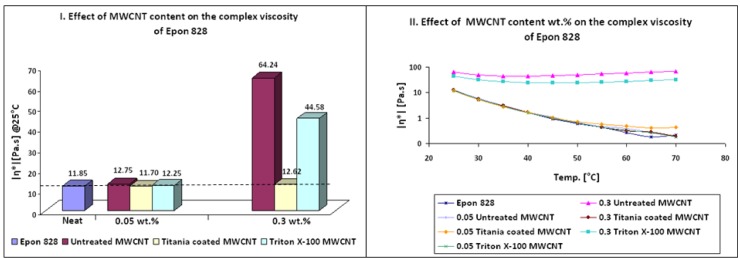
Effect of MWCNT content on the complex viscosity of Epon 828 before curing.

Results of complex viscosity |η*| of Epon 828/MWCNT suspensions before curing were obtained by Dynamic Parallel Plate Rheometry and can be seen in Table 5 and Figure 1. It can be noticed that addition of 0.05 wt % untreated or treated MWCNT did not change significantly the complex viscosity of Epon 828 at the temperature range tested. At higher contents of MWCNT only titania coated MWCNT suspensions maintained the low viscosity of neat epoxy matrix. According to Abdalla *et al.* [17] higher viscosity indicates of either a better dispersed system or stronger interfacial interactions. Conversely, Song *et al.* [64], claimed that poorly dispersed CNT in epoxy resin have a more solid-like behavior and leads to higher complex viscosity. In the current study, at 0.3 wt % MWCNT loading, untreated MWCNTs exhibited the highest complex viscosity and the lowest thermo-mechanical properties after curing. These findings can only suggest that untreated MWCNTs lead to a relatively poor dispersion, while the titania coated MWCNTs lead to better dispersion. In addition, it can be assumed that titania coating reduced the VDW interaction between MWCNTs themselves by neutralizing the polar groups known to be present at MWCNT surface. In order to maintain the low viscosity the content of untreated and Triton X-100 treated MWCNTs should be lower than 0.3 wt %.

**Table 5:** Complex viscosity |η*| of Epon 828/MWCNT suspensions before curing.

Sample	Additive (wt %)	Complex viscosity (Pa.s) @25 °C	Complex viscosity variation (%)
Untreated MWCNT	0.05	12.75	8
	0.30	64.24	442
Titania coated MWCNT	**0.05**	**11.70**	**−1**
	**0.30**	**12.62**	**6**
Triton X-100 MWCNT	0.05	12.25	3
	0.30	44.58	276

**Figure 2:**
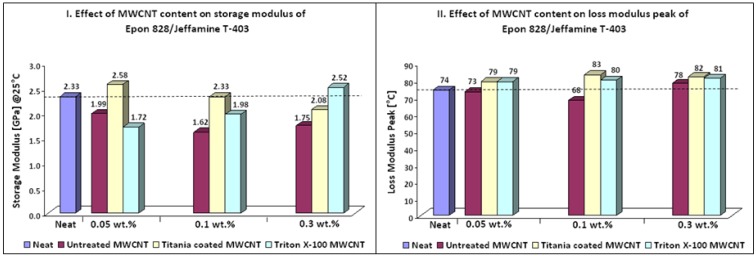
Effect of MWCNT content on thermo-mechanical properties of nanocomposites (Epoxy system 1).

**Figure 3:**
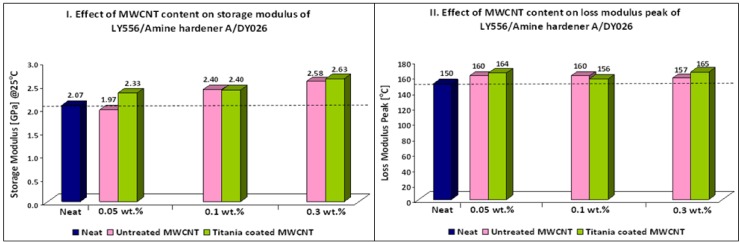
Effect of MWCNT content on thermo-mechanical properties of nanocomposites (Epoxy system 2).

Thermo-mechanical properties of Epon 828/Jeffamine T-403 nanocomposites are depicted in Table 6 and Figure 2. It can be seen that addition of treated MWCNTs caused a higher crosslink density compared to neat resin, as could be noticed by the increase of approximately 10% in the Tg of the nanocomposites. Furthermore, treated MWCNT induced an increase in thermal stability of nanocomposites in the rubbery state of storage modulus (Figure 3 b,e). According to Ma *et al.* [36] this behavior can be explained in terms of interfacial bonding. The improved interfacial bonding reduces the mobility of the matrix around the MWCNT, increasing thermal stability at elevated temperatures. Simultaneous increase of approximately10% in storage modulus and Tg can be seen for 0.05 wt % titania coated MWCNT and 0.3 wt % Triton X-100 MWCNT. Nanocomposites containing titania coated MWCNT showed the best thermo-mechanical properties. This finding can be due to the hydrophilic and polar nature of titania. Another explanation can be the possible chemical reaction between OH groups known present on CNTs surface and titanium precursor molecules [42]. It should be mentioned that the addition of treated MWCNT did not lead to toughening effects.

**Table 6:** Epoxy system 1: Thermo-mechanical properties of nanocomposites.

Sample	Additive (wt %)	Storage modulus (GPa) @25 °C	Storage modulus variation (%)	Loss modulus peak (°C)	Loss modulus peak variation (%)
Epon 828/Jeffamine T-403	0	2.33		74	
Untreated MWCNT	0.05	1.99	−15	73	−1
	0.10	1.62	−30	68	−8
	0.30	1.75	−25	78	5
Titania Coated MWCNT	0.05	2.58	11	79	7
	0.10	2.33	0	83	12
	0.30	2.08	−11	82	11
Triton X-100 MWCNT	0.05	1.72	−26	79	7
	0.10	1.98	−15	80	8
	0.30	2.52	8	81	9

Table 7 and Figure 3 show the thermo-mechanical properties of LY556/Amine hardener A/DY026 nanocomposites. An increase in the Tg of the samples containing titania coated and untreated MWCNTs was noticed, most probably as a result of a higher crosslink density. Moreover, an increase in storage modulus has been also observed through the whole temperature range investigated. Maximum increase of 10% in Tg and 30% in storage modulus was achieved for nanocomposites containing 0.3 wt % titania coated MWCNTs. The unexpected improvement in thermo-mechanical properties of nanocomposites containing untreated MWCNTs can be explained by the relatively low viscosity of LY556 resin. This characteristic imparts low shear forces during sonication resulting in less damage to the MWCNT structure. In addition, a relatively low viscosity of the matrix before curing favors the formation of uniform nanocomposites [16]. These explanations can also relate to the better results obtained for nanocomposites containing titania coated MWCNTs of epoxy system 2 compared to epoxy system 1. It should be mentioned that toughening effects have not been observed.

**Table 7:** Epoxy system 2: Thermo-mechanical properties of nanocomposites.

Sample	Additive (wt %)	Storage modulus (GPa) @25 °C	Storage modulus variation (%)	Loss modulus peak (°C)	Loss modulus peak variation (%)
LY556/Amine hardener A/DY026	0	2.07		150	
Untreated MWCNT	0.05	1.97	-5	160	7
	0.10	2.40	16	160	7
	0.30	2.58	25	157	5
Titania Coated MWCNT	0.05	2.33	13	164	9
	0.10	2.40	16	156	4
	0.30	2.63	27	165	10

**Figure 4:**
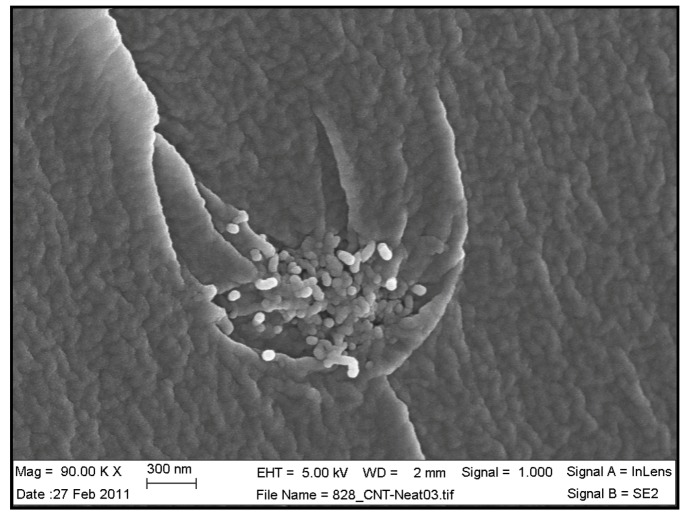
SEM image of epoxy system 1 with 0.3% untreated MWCNT showing a “crack front bowing” toughening mechanism.

Figure 4 and Table 8 show SEM and AFM images of fractured surface morphologies of nanocomposites contain 0.3 wt % MWCNT based on epoxy systems 1 and 2. Nanocomposites containing titania coated MWCNT and uncoated MWCNT have shown a fracture mechanism known as crack front bowing, that can clearly be seen from SEM image in Figure 4. From AFM images (Table 8) a crack pinning mechanisms was noticed for the titania coated MWCNT nanocomposites, a toughening mechanism that indicates the strong interaction between the carbon nanotubes and the epoxy matrix through the coating. The toughening effect could not be clearly seen in the thermo-mechanical results due to the poor dispersion in the matrix, in all cases.

**Table 8:** AFM images of fractured surface morphologies of nanocomposites contain 0.3 wt% MWCNT.

AFM images (contact mode; 5 × 5 µm)		Sample	Epoxy System *
3D	2D		
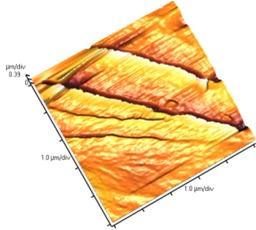	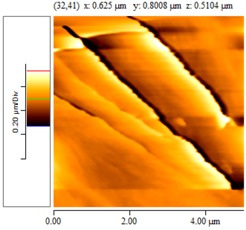	Neat	1
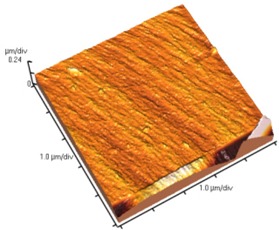	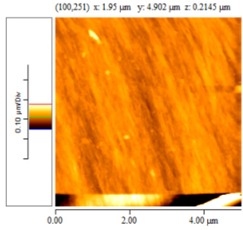	Untreated MWCNT	
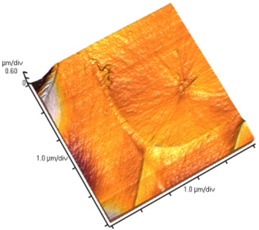	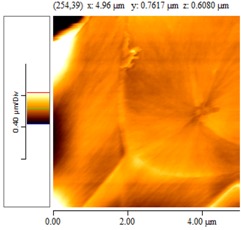	Titania coated MWCNT	
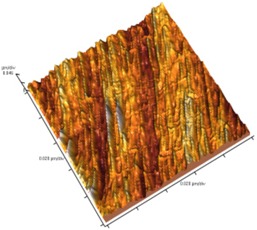	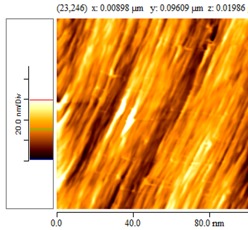	Neat	2
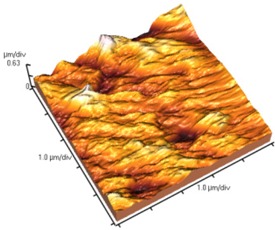	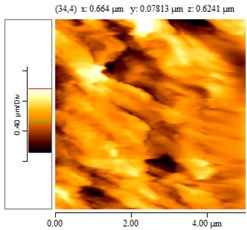	Untreated MWCNT	

* Epoxy system 1: Epon 828/Jeffamine T-403; Epoxy system 2: LY556/Amine hardener A/DY026.

**Figure 5:**
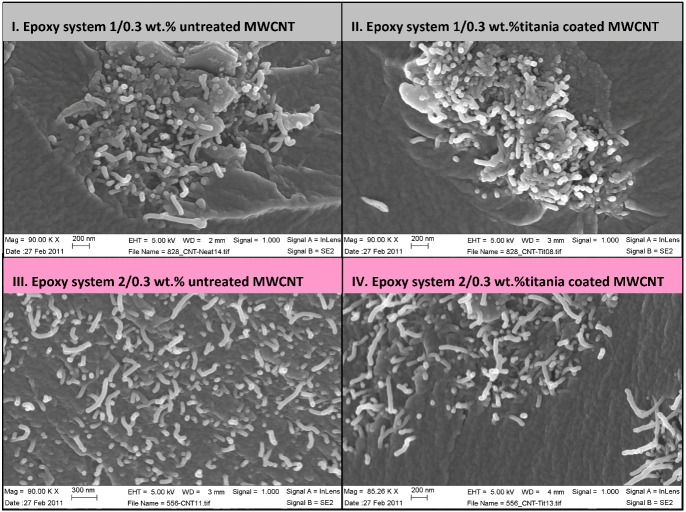
SEM images of titania coated and non-treated MWCNT nanocomposites.

Figure 5 depicts selected scanning electron microscopy images of surface fractured nanocomposites (cryogenic fracture) showing higher amounts of broken nanotubes in epoxy system 1 for both untreated and titania coated MWCNT samples. Broken nanotubes at the fracture surface indicate a strong interfacial strength. Longer nanotubes could be seen in SEM images of the epoxy system 2, indicating a pull-out mechanism, related to a weaker nanotube/matrix interface. According Gojny *et al.* [4] a fracture of the outer layer and a telescopic pull-out of the inner tube(s) can occur due to strong interfacial bonding. Accordingly it is possible that some images of pulled-out MWCNTs in Figure 5 represent a nanotube inner layer pull-out, implying on a strong interfacial bonding.

## 4. Conclusions

It can be concluded from the experimental results that titania coating obtained from a sol-gel method is a preferable surface treatment for enhancement of thermo-mechanical properties of epoxy nanocomposites, without causing a significant increase in viscosity, a crucial parameter for filament winding resin systems. A maximum increase of about 10% in Tg has been achieved for the two epoxy matrices containing 0.05-0.3 wt % titania coated MWCNT. An increase of 30% in the storage modulus has been achieved for LY556/Amine hardener A/DY026 matrix containing 0.3 wt % titania coated MWCNT.
